# A novel gain‐of‐function *STAT3* variant in infantile‐onset diabetes associated with multiorgan autoimmunity

**DOI:** 10.1002/mgg3.2407

**Published:** 2024-02-26

**Authors:** Qiaoli Zhou, Dandan Chen, Jing Yu, Bixia Zheng, Wei Zhou, Zhanjun Jia, Aihua Zhang, Wei Gu

**Affiliations:** ^1^ Department of Endocrinology Children's Hospital of Nanjing Medical University Nanjing China; ^2^ Department of Child Healthcare Lianyungang Maternal and Children's Hospital Lianyungang China; ^3^ Nanjing Key Laboratory of Pediatrics Children's Hospital of Nanjing Medical University Nanjing China

**Keywords:** diabetes, gain‐of‐function, multiorgan autoimmunity, *STAT3* gene

## Abstract

**Background:**

Germline gain‐of‐function (GOF) variants in the signal transducer and activator of transcription 3 (*STAT3*) gene lead to a rare inherited disorder characterized by early‐onset multiorgan autoimmunity.

**Methods:**

We described a Chinese patient with infantile‐onset diabetes and multiorgan autoimmunity. The patient presented with early‐onset type 1 diabetes and autoimmune hypothyroidism at 7 months. During the 7.5‐year follow‐up, she developed pseudo‐celiac enteropathy at 1 year of age and showed severe growth retardation. Whole‐exome sequencing was performed and the novel variant was further assessed by in vitro functional assays.

**Results:**

Whole‐exome sequencing revealed a novel variant (c.1069G>A, p.Glu357Lys) in the DNA‐binding domain of STAT3. In vitro functional studies revealed that p.Glu357Lys was a GOF variant by increasing *STAT3* transcriptional activity and phosphorylation. In addition, the *STAT3* Glu357Lys variant caused dysregulation of insulin gene expression by enhancing transcriptional inhibition of the insulin gene enhancer binding protein factor 1 (*ISL1*).

**Conclusion:**

In the current study, we describe clinical manifestations and identify a novel *STAT3* GOF variant (c.1069G>A) in a Chinese patient. This activating variant impairs insulin expression by increasing transcriptional inhibition of its downstream transcription factor *ISL1*, which could be involved in the pathogenesis of early‐onset diabetes.

## Graphical abstract

Gain‐of‐function (GOF) variants of *STAT3* cause early‐onset multiorgan autoimmunity. In this study, we described a patient with early‐onset diabetes, hypothyroidism, enteropathy, and postnatal short stature. Of note, the chronic diarrhea lasted 3 years and resolved spontaneously. We identified a novel *STAT3* GOF variant p.E357K. Further functional data showed that the variant caused dysregulation of insulin gene expression through inhibition of the transcription factor *ISL1*.

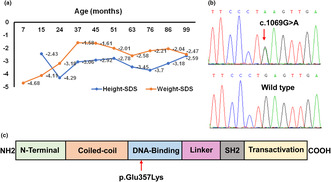

## 1 INTRODUCTION

Polyautoimmunity is defined as the presence of more than one autoimmune disease in a single patient (Anaya, 2014). The mechanisms by which polyautoimmunity develops are usually multifactorial, including a combination of genetic, immunological, and environmental factors. Single gene variants that result in severe autoimmunity and immune dysfunction have been described. Relevant genes include *AIRE*, *FOXP3*, *IL2RA*, *LRBA*, *STAT1*, *STAT5B*, *CTLA‐4*, and *ITCH* (Amaya‐Uribe et al., 2019; Azizi et al., 2018). The signal transducer and activator of transcription 3 (*STAT3)* (OMIM:102582) are involved in inflammation, tissue regeneration, cellular proliferation, survival, and differentiation. The signaling pathway involved in STAT3 pathogenesis is the JAK–STAT pathway. A cytokine, such as IL‐6, binds to its receptor, which leads to the phosphorylation of Janus Kinases (JAKs) (Morris et al., 2018). These activated JAKs then phosphorylate STAT3, allowing it to form dimers and translocate to the nucleus, where it acts as a transcription factor. Germline loss‐of‐function (LOF) variants in the *STAT3* gene result in autosomal‐dominant hyperimmunoglobulin‐E syndrome (OMIM:147060) which is characterized by high serum IgE, eosinophilia, eczema, and immunodeficiency (Woellner et al., 2010). In addition, germline gain‐of‐function (GOF) variants in *STAT3* have recently been associated with an extremely rare autosomal‐dominant early‐onset multiorgan autoimmunity (OMIM: 615952), with a median age at onset of 3 years (Flanagan et al., 2014). The phenotype of *STAT3* GOF variant is characterized by lymphadenopathy with hepatosplenomegaly, postnatal growth retardation, immunodeficiency, and autoimmune disorders, including cytopenia, enteropathy, diabetes, thyroiditis, arthritis, atopic dermatitis, and interstitial lung disease (Fabre et al., 2018; Fabre et al., 2019; Giovannini‐Chami et al., 2019). Activating germline variants in *STAT3* impair the development of regulatory T cells and promote the expansion and activation of T helper type 17 (TH17) cells. The dysregulation of TH17 cells is thought to play a critical role in autoimmune diseases, including type 1 diabetes (Flanagan et al., 2014). Early‐onset diabetes is the most frequent endocrine manifestation of multiorgan autoimmunity caused by *STAT3* GOF variants. While *STAT3* GOF variants have been shown to lead to early‐onset diabetes by affecting the regulation of immune‐related cells, recent studies demonstrate that activating variants in *STAT3* can also affect insulin secretion, pancreatic β‐cell function, and pancreatic development (Kostromina et al., 2010; Saarimaki‐Vire et al., 2017; Velayos et al., 2017).

To date, a total of 72 GOF variants of the *STAT3* gene have been reported in less than 200 patients in the published literature (Leiding et al., 2023). In the present study, we reported a new case with *STAT3* GOF phenotype suffering from infantile‐onset autoimmune diabetes and hypothyroidism at 7 months, chronic diarrhea from 1 year of age, and postnatal short stature. Whole‐exome sequencing identified a novel GOF variant (NM_139276.2, c.1069G>A, p.Glu357Lys) in the *STAT3* gene. Moreover, functional data showed that the *STAT3* p.Glu357Lys variant caused dysregulation of insulin gene expression through inhibition of the transcription factor, insulin gene enhancer binding protein factor 1 (*ISL1*).

## 2 MATERIALS AND METHODS

### 2.1 Whole‐exome sequencing and bioinformatics analysis

Whole‐exome sequencing of the patient's DNA samples was performed in order to identify any genetic cause of multiorgan autoimmunity. Genomic DNA was isolated from whole blood using a DNA isolation kit (Tiangen, China) according to the manufacturer's instructions. 50–100 ng of genomic DNA was subjected to enriched library preparation on an Illumina HiSeq X Ten (Illumina, USA) platform. Low‐quality variations of the quality score, < 20 (Q20), were filtered out. Sequencing reads were mapped to the GRCh37/Hg19 reference genome using Burrows‐Wheeler Aligner software. All variants identified were annotated using the 1000 Genomes database, dbSNP, Genome Aggregation Database, and ExAC database. Variants located within exons, introns, predictive splice sites, and UTRs were identified with a minor allele frequency <0.05. Candidate variants were evaluated using the American College of Medical Genetics and Genomics criteria (ACMG) and further validated by direct Sanger sequencing to confirm the identities of the variants. SIFT (https://sift.bii.a‐star. edu.sg/), PolyPhen‐2 (http://genetics.bwh.harvard.edu/pph2/), Mutation Taster (http://www.mutationtaster.org) and Provean (http://provean.jcvi.org/seq_submit.php) were performed to evaluate the possible functional significance of the identified variants.

### 2.2 Construction of expression plasmids

Wild‐type full‐length human *STAT3* cDNA was purchased and cloned into a pcDNA3.1 vector using a Clon Express Entry one‐step cloning kit (Vazyme, China). Site‐directed mutagenesis was performed to introduce the Glu357Lys variant into the pcDNA3.1 plasmid containing the wild‐type *STAT3* cDNA using the PCR‐based DpnI‐treatment method (Vazyme). Targeted sequences were confirmed by a dideoxy‐nucleotide sequencing method.

### 2.3 Cell culture and transfection

Human embryonic kidney (HEK)‐293 cells or mouse MIN6 pancreatic β‐cells were transiently transfected with wild‐type or mutant *STAT3* plasmids. HEK‐293 cells were grown in DMEM supplemented with 10% fetal bovine serum at 37°C in an atmosphere of 5% CO_2_. MIN6 cells were cultured in DMEM supplemented with 15% FBS and 50 μmol/L β‐mercaptoethanol at 37°C in a humidified atmosphere with 5% CO_2_. At 50%–70% confluence, HEK‐293 or MIN6 cells were transfected with purified plasmids (containing either wild‐type or mutant *STAT3*) using Lipofectamine 2000 (Invitrogen, America).

### 2.4 Western blot analysis

The total STAT3 and phosphorylated STAT3 (p‐STAT3) were measured by western blot with and without interleukin‐6 (IL‐6) stimulation. Western blotting was performed using mouse monoclonal anti‐STAT3 antibodies (Cell Signaling Technology, Cat# 9139 T) and rabbit monoclonal p‐STAT3 antibodies (Cell Signaling Technology, Cat# 9145 T). Wild‐type or mutant STAT3 plasmids were transfected into MIN6 cells. For phosphorylation assays, MIN6 cells were treated with recombinant mouse IL‐6 (Novoprotein, China) at a concentration of 20 ng/mL for 12 h. Forty micrograms of total protein was resolved by 10% SDS‐PAGE before transfer to PVDF membranes. Membranes were visualized by chemiluminescence reaction. Band intensity was quantified using ImageJ software (National Institutes of Health, Bethesda, MD).

### 2.5 Luciferase reporter assay

HEK‐293 cells were seeded in 24‐well plates and transiently transfected using Lipofectamine. The *STAT3*‐responsive reporter plasmid (pGL3‐*STAT3*‐Luc) (Haixing Biological Technology Co., Ltd, Suzhu, China) was used to detect *STAT3* activation. The promoter sequence of the *ISL1* gene was amplified by PCR and inserted into a pGL3‐basic cloning vector to construct a recombinant luciferase reporter plasmid, pGL3‐*ISL1*‐Promotor‐luc (Haixing Biological Technology Co., Ltd, Suzhu, China). 0.5 μg pGL3‐*STAT3*‐Luc or pGL3‐*ISL1*‐Promotor‐luc was co‐transfected with 0.25 μg wild‐type *STAT3*‐pcDNA3.1 or 0.25 μg mutant *STAT3*‐pcDNA3.1 using Lipofectamine, respectively. A Renilla luciferase reporter gene was used as the internal control vector. The firefly luciferase and Renilla luciferase activities were assayed using the Dua‐Luciferase Reporter Assay Kit (E1910; Promega, Madison, WI) in accordance with the manufacturer's protocols. The firefly luciferase activity was normalized to Renilla luciferase activity.

### 2.6 Quantitative real‐time PCR

Total RNA was isolated from MIN6 cells transfected with wild‐type and mutant *STAT3*‐pcDNA3.1 expression plasmids using TRIzol Reagent (Takara, Japan) according to the manufacturer's protocol. cDNA was reverse transcribed from 1 μg total RNA using a SuperScript III PT‐PCR kit (Invitrogen, America) with random hexanucleotide primers. Expression of *ISL1*, *INS‐1*, and *INS‐2* were assayed by real‐time PCR with SYBR Green using the primer pair sequences listed in Supplementary Table S1. PCR reactions were run in triplicate and GAPDH was used as an internal control to normalize expression levels.

## 3 RESULTS

### 3.1 Clinical characteristics of the proband

**FIGURE 1:**
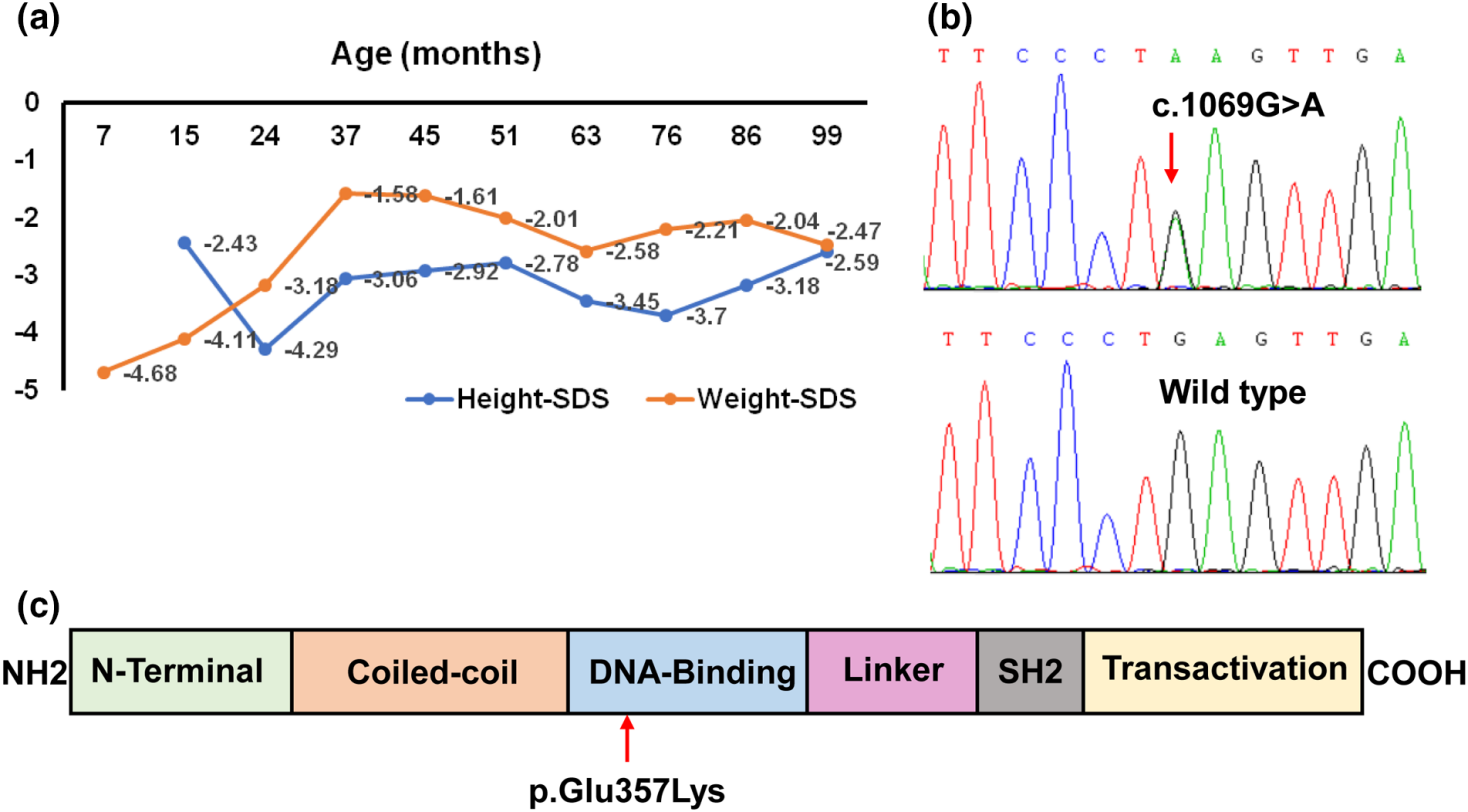
The growth curve and *STAT3* gene variant of the patient. (a) Changes in height and weight *Z* scores of the patient from the time of the first presentation to the most recent follow‐up visit to the clinic. (b) The missense variant (NM_139276.2, c.1069G>A), which predicted a glutamic acid to lysine substitution at codon 357 of STAT3, was identified in the patient, which was not detected in her mother and father. (c) The position of the de novo variant p.Glu357Lysis shown below the STAT3 domain.

The patient was the first child born to non‐consanguineous, healthy parents after an uneventful pregnancy and delivery. She had normal birth weight and length, 3.35 kg and 50 cm, respectively. At 7 months, she was diagnosed with type 1 diabetes mellitus (positive islet cell and glutamic acid decarboxylase antibodies) and autoimmune hypothyroidism (positive thyroid peroxidase and antithyroglobulin antibodies). At 1 year of age, the patient developed watery, non‐bloody diarrhea (5–6 episodes per day). A stool sample was negative for occult blood, leukocytes, ova, or parasites. The results of multiple stool cultures were negative. The immunological evaluation showed normal IgG, IgM, IgE, elevated IgA, normal CD3+, CD4+, CD8+ T‐cells and NK cells, and decreased B‐cells. She had no response to a gluten‐free diet and peptide‐based enteral formula and needed daily oral potassium supplementation to maintain serum potassium within the normal range. From the age of 2 years, diarrhea gradually improved until it ceased at the age of 4 years when oral potassium supplementation could be stopped due to normal serum concentrations being achieved. The patient received levothyroxine and insulin treatment from 7 months of age. During the 7.5‐year follow‐up, her daily insulin requirement ranged from 0.5 to 1 U/kg/d, and a relatively high dose of levothyroxine, from 50 to 150 μg/d, was needed to maintain TSH and FT4 within normal ranges. Additionally, she evinced profound growth retardation as can be seen from her growth curve (Figure 1a). Assessment of the growth hormone axis was not conducted and the patient did not receive treatment with growth hormone for severe short stature due to the presence of diabetes. Tofacitinib was recommended to the patient's parents, but they refused because of concerns about side effects.

### 3.2 Genetic findings and pathogenicity analysis

A de novo, heterozygous variant c.1069G>A in the *STAT3* gene (GenBank reference sequence NM_139276.2) was identified by whole‐exome sequencing and confirmed by Sanger sequencing (Figure 1b). This missense variant has not been reported in published literature or the Human Genome Mutation Database. The c.1069G>A variant located in exon 11, predicted a glutamic acid to lysine substitution at codon 357 of STAT3 (p.Glu357Lys). The glutamic acid at codon 357 maps to the DNA‐binding domain of STAT3 (Figure 1c) and is highly conserved among different species. The missense variant p.Glu357Lys was predicted to have a severe effect on protein function (SIFT: 0, PolyPhen2: 1, Mutation Taster: “disease causing,” Provean: “deleterious”). Based on the guidelines from the ACMG, the variant was rated as likely‐pathogenic (PS2 + PM2_Supporting + PP3 + PP4).

### 3.3 Expression of STAT3/p‐STAT3 proteins in MIN6 cells

**FIGURE 2:**
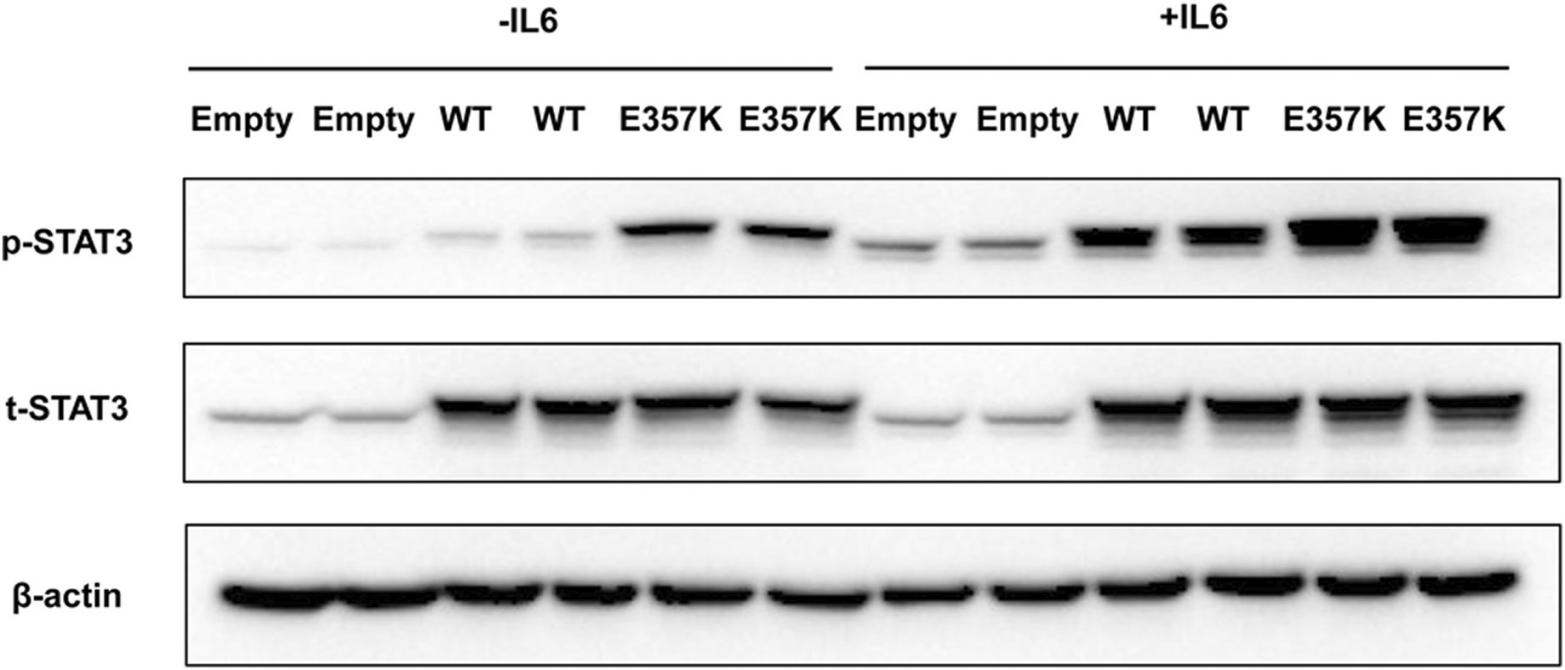
Total and phosphorylated STAT3 expression. Western blot analysis of the total STAT3 and phosphorylated STAT3 expression in the wild‐type (WT) and mutant STAT3 in MIN6 cells.

Under basal and IL‐6 treatment conditions, levels of total STAT3 protein did not differ significantly between the cells transfected with plasmids overexpressing wild‐type and mutant *STAT3* (Figure 2). Without IL‐6 stimulation, the expression of p‐STAT3 in the wild‐type STAT3 was weak, while p‐STAT3 was significantly increased in the mutant p.Glu357Lys. Stimulation with IL‐6 (20 ng/mL) led to an increase in STAT3 phosphorylation in wild‐type and mutant *STAT3*‐transfected cells, while phosphorylation levels were more abundant in the mutant p.Glu357Lys. (Figure 2).

### 3.4 Luciferase reporter assay and quantitative real‐time PCR

**FIGURE 3:**
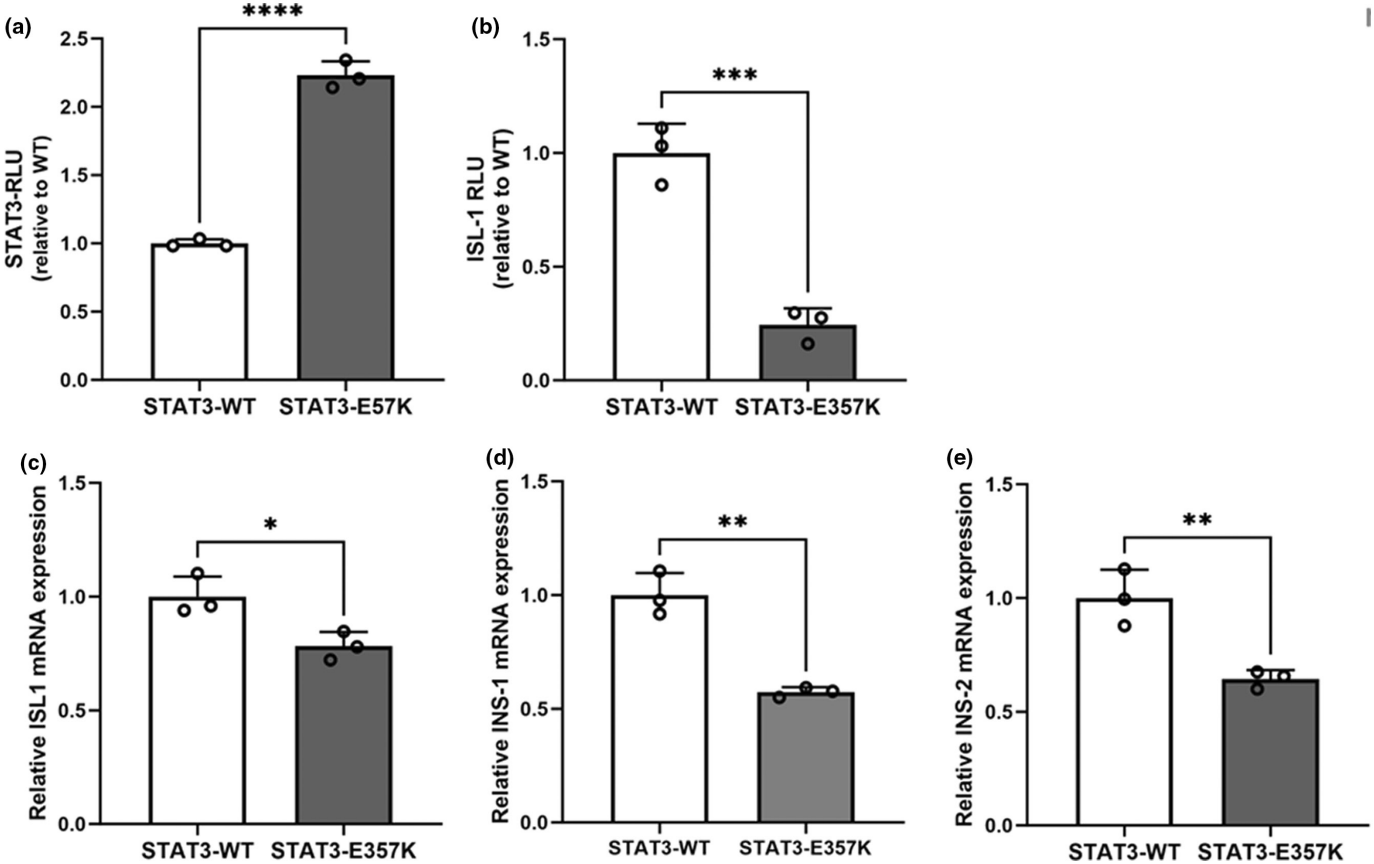
Functional characterization of the *STAT3*‐Glu357Lys (*STAT3*‐E357K) variant in the *STAT3* transcriptional activity. (a,b) Luciferase reporter assay. *STAT3* transcriptional activity (a) and ISL1 promoter activity (b) in HEK‐293 cells after transfection with expression vector *STAT3*‐WT and *STAT3*‐E357K. All the experiments were normalized to a Renilla internal control vector. (c–e) Quantitative real‐time PCR. mRNA expression of *ISL1*(c), *INS‐1*(d), and *INS‐2*(e) was assayed by real‐time PCR and normalized to the housekeeping gene, GAPDH, in MIN6 β‐cells after transfection with the expression vectors, *STAT3*‐WT and *STAT3*‐E357K. ****p < 0.001; **p < 0.005; *p < 0.05; mean ± SEM, 95%CI; unpaired Student's *t*‐test.

Under the basal condition, the mutant p.Glu357Lys conferred significantly increased *STAT3* transcriptional activity compared with the wild‐type in *STAT3*‐luciferase reporter assay (Figure 3a). Luciferase reporter assay showed the transcriptional activity of ISL1 promoter was decreased in the mutant compared with the wild‐type (Figure 3b). Quantitative real‐time PCR showed the expression levels of *ISL1*, *INS‐1*, and *INS‐2* were severely decreased in MIN6 cells transiently expressing the mutant *STAT3* compared with the wild‐type *STAT3* (Figure 3c–e).

## 4 DISCUSSION

In the present study, the patient displayed prominent features of early‐onset multiorgan autoimmunity cased by *STAT3* GOF variants, including infantile‐onset endocrinopathies, enteropathy, and postnatal short stature. Of note, the chronic diarrhea lasted 3 years and resolved spontaneously. Genetic analysis showed a de novo heterozygous missense *STAT3* variant (c.1069A>G) causing glutamine‐to‐lysine substitution at position 357.

The *STAT3* gene is located on chromosome 17q21, comprises 24 exons, and encodes a 770 amino acid protein belonging to the STAT family of transcription factors. The STAT3 protein comprises the following domains: N‐terminal domain coiled‐coil domain, DNA‐binding domain, linker, Src‐homology 2 (SH2), and transactivation domain (Hirano et al., 2000). Previous studies have shown that *STAT3* variants from patients with early‐onset multiorgan autoimmunity are mostly mapped to the DNA‐binding domain. The novel *STAT3* variant p.Glu357Lys which is also located in the DNA‐binding domain is thought to increase *STAT3* transcriptional activity resulting in a GOF phenotype. Consistently, our functional analysis also indicated the p.Glu357Lys variant increased the phosphorylation and transcriptional activity of the STAT3.

Diabetes was the earliest endocrine manifestation in early‐onset multiorgan autoimmunity cased by *STAT3* GOF variants. A further 10 patients with *STAT3* GOF phenotype have been reported to have early‐onset diabetes (Flanagan et al., 2014; Milner et al., 2015; Nabhani et al., 2017; Sediva et al., 2017; Velayos et al., 2017). Diabetes in seven of these individuals presented before 12 months and was treated with insulin from diagnosis (Flanagan et al., 2014; Milner et al., 2015; Sediva et al., 2017; Velayos et al., 2017). As with our case, the majority of these cases had positive islet autoantibodies. Based on the strong immunological phenotype, diabetes was presumed to be the result of an autoimmune attack on the pancreatic β‐cells (Fabbri et al., 2019; Harris et al., 2007). Notably, a reported case, attributed to the *STAT3* GOF variant p.Pro330Ser, manifested neonatal diabetes accompanied by detectable islet autoantibodies, autoimmune hypothyroidism, and enteropathy (Velayos et al., 2017). Functional analysis demonstrated that the p.Pro330Ser variant led to aberrant activation of STAT3, resulting in deleterious effects on pancreatic β‐cells. The functional results of our study were in line with it. The studies demonstrated that the identified GOF variants (p.Pro330Ser; p.Glu357Lys) led to hyper‐inhibition of *ISL1* and consequently to a decrease in insulin gene expression in a rat insulinoma cell line. In addition, *STAT3* is involved pancreatic differentiation in patient‐derived induced pluripotent stem cells (iPSCs) derived from a neonatal diabetes patient with a GOF STAT3 variant p.Lys392Arg (Saarimaki‐Vire et al., 2017). β‐cell‐specific *STAT3* knockout mice exhibit insulin secretory defects and impaired pancreatic islet architecture (Gorogawa et al., 2004). Thus, in addition to causing dysregulation of immune‐related cells, *STAT3* GOF variants may be implicated in diabetes by altering the development and function of pancreatic β‐cells. To date, the few studies conducted has been limited to individuals with diabetes mellitus who have a specific missense variant in the *STAT3* gene, and the functional validation of human pancreatic β‐cells is lacking. The impact of *STAT3* GOF variants on diabetes outside of the immune system has yet to be investigated further in human pancreatic β‐cells.

As shown in Figure 1, the height of the patient decreased severely after 1 year owing to protracted diarrhea. Although her height started to increase after 2 years due to improved diarrhea, she still could not achieve normal height velocity probably arising from the insufficient growth hormone after 4 years. In a majority of patients with *STAT3* GOF variants, profound growth retardation is exhibited and this may be related to gastrointestinal disease, diabetes, hypothyroidism, recurrent infections, or immunosuppressive treatment. Similar to reported cases, despite good control of the enteropathy, diabetes, and hypothyroidism, our patient failed to reach a normal growth velocity (Sediva et al., 2017). A recent study has shown that *STAT3* GOF variants disrupt the growth hormone signaling pathway by reducing the transcriptional activity of *STAT5B* (Gutierrez et al., 2018). In consequence, postnatal growth retardation could be inherent to the *STAT3* GOF variants.

A previously reported variant p.Val353Phe, which also mapped to the DNA‐binding domain, near to the novel variant p.Glu357Lys, was identified in a 13‐year‐old boy with clinical features of autoimmune cytopenia (hemolytic anemia, thrombocytopenia, and neutropenia), lymphoproliferation and inflammatory lung disease. He showed no endocrinopathies, enteropathy, and postnatal short stature (Milner et al., 2015). Members of the same family carrying the identical *STAT3* GOF variants (p.Ala703Thr, p.Thr716Met) had diverse clinical phenotypes and incomplete penetrance (Milner et al., 2015; Tanita et al., 2021). Furthermore, phenotypic characteristics of early‐onset multiorgan autoimmunity cased by *STAT3* GOF variants overlap with other monogenic autoimmune disorders. Therefore, establishing a molecular diagnosis and long‐term follow‐up is critical for patients with *STAT3* GOF variants. Autoimmune symptoms of *STAT3* GOF patients have been treated with a broad spectrum of immunosuppressive medications and targeted drugs in published case reports. The response to immunosuppressive medications such as systemic steroids and mycophenolate mofetil, varied among individuals, and some patients showed no significant benefit from these drugs (Giovannini‐Chami et al., 2019; Khoury et al., 2017; Russell et al., 2018). Targeted drug therapy has shown promise in the treatment of *STAT3* GOF patients. Drugs targeting the JAK–STAT signaling pathway have been used and may be beneficial for patients with STAT3 *GOF* variants (Forbes et al., 2018; Sarfati et al., 2021). However, further research and clinical studies are needed to evaluate the efficacy and safety of the targeted therapy. In order to improve outcomes, tofacitinib, a JAKs inhibitor, was suggested to our patient. Unfortunately, the child did not receive the recommended treatment due to parents' reluctance.

## 5 CONCLUSION

In summary, we describe a patient with infantile‐onset diabetes and multiorgan autoimmunity caused by a novel germline GOF variant (c.1069A>G) in *STAT3*. This activating *STAT3* variant results in the decrease of insulin expression by repressing the transcriptional activity of *ISL1*, which could be involved in the pathogenesis of pancreatic β‐cell dysfunction and early‐onset diabetes. Patients with early‐onset multiorgan autoimmunity cased by *STAT3* GOF variants exhibit clinical heterogeneity with incomplete penetrance and have overlapping features with other monogenic, multiorgan, autoimmune disorders. Therefore, we highlighted the importance of establishing a specific genetic diagnosis.

## AUTHOR CONTRIBUTIONS

Qiaoli Zhou and Dandan Chen collected and analyzed the clinical data. Qiaoli Zhou and Jing Yu performed the experimentation. Bixia Zheng and Wei Zhou performed whole‐exome sequencing analysis and interpreted the genetic results. Qiaoli Zhou, Jing Yu, and Zhanjun Jia analyzed the experimental data. Aihua Zhang and Wei Gu conceived the study and participated in its design and coordination. Qiaoli Zhou, Aihua Zhang, and Wei Gu wrote and edited the manuscript. All authors have read and approved the final manuscript.

## ETHICS STATEMENT

The current study was approved by the ethics committee of the Children’s Hospital of Nanjing Medical University. At the age of 7 months, the proband was admitted to the Department of Endocrinology, Children’s Hospital of Nanjing Medical University due to early‐onset type 1 diabetes and autoimmune hypothyroidism. Clinical data was collected and reviewed. Peripheral blood samples from the proband and her parents were collected for the genetic screen.

## Supporting information


Supplementary Table 1.

## Data Availability

The data that support the findings of this study are available from the corresponding author upon reasonable request.
