# Supplementary material for: A novel gain‐of‐function STAT3 variant in infantile‐onset diabetes associated with multiorgan autoimmunity
Source: Mol Genet Genomic Med. 2024 Feb 26;12(2):e2407. doi: 10.1002/mgg3.2407 (PMC10895381; doi:10.1002/mgg3.2407)
Supplement: Supplementary file 1 — Supplementary Table 1. [file MGG3-12-e2407-s001.docx]

| **Supplementary Table 1.**The primer pair sequences for qPCR | |
| --- | --- |
| Primers | Sequence |
| ISL1-Forward | TTTCCCTGTGTGTTGGTTGC |
| ISL1-Reverse | TGATTACACTCCGCACATTTCA |
| INS-1-Forward | CACTTCCTACCCCTGCTGG |
| INS-1-Reverse | ACCACAAAGATGCTGTTTGACA |
| INS-2-Forward | GCTTCTTCTACACACCCATGTC |
| INS-2-Reverse | AGCACTGATCTACAATGCCAC |
